# On the Corrosion Fatigue of Magnesium Alloys Aimed at Biomedical Applications: New Insights from the Influence of Testing Frequency and Surface Modification of the Alloy ZK60

**DOI:** 10.3390/ma15020567

**Published:** 2022-01-12

**Authors:** Mikhail Linderov, Alexander Brilevsky, Dmitry Merson, Alexei Danyuk, Alexei Vinogradov

**Affiliations:** 1 Institute of Advanced Technologies, Togliatti State University, 445020 Togliatti, Russia; dartvi@gmail.com (M.L.); alexandrbril@yandex.ru (A.B.); D.Merson@tltsu.ru (D.M.); alexey.danyuk@gmail.com (A.D.); 2 Department of Mechanical and Industrial Engineering, Norwegian University of Science and Technology, 4791 Trondheim, Norway

**Keywords:** magnesium alloys, fatigue, corrosion, corrosion fatigue, coatings, frequency, stress corrosion cracking

## Abstract

Magnesium alloys are contemporary candidates for many structural applications of which medical applications, such as bioresorbable implants, are of significant interest to the community and a challenge to materials scientists. The generally poor resistance of magnesium alloys to environmentally assisted fracture, resulting, in particular, in faster-than-desired bio-corrosion degradation in body fluids, strongly impedes their broad uptake in clinical practice. Since temporary structures implanted to support osteosynthesis or healing tissues may experience variable loading, the resistance to bio-corrosion fatigue is a critical issue that has yet to be understood in order to maintain the structural integrity and to prevent the premature failure of implants. In the present communication, we address several aspects of the corrosion fatigue behaviour of magnesium alloys, using the popular commercial ZK60 Mg-Zn-Zr alloy as a representative example. Specifically, the effects of the testing frequency, surface roughness and metallic coatings are discussed in conjunction with the fatigue fractography after the testing of miniature specimens in air and simulated body fluid. It is demonstrated that accelerated environmentally assisted degradation under cyclic loading occurs due to a complicated interplay between corrosion damage, stress corrosion cracking and cyclic loads. The occurrence of corrosion fatigue in Mg alloys is exaggerated by the significant sensitivity to the testing frequency. The fatigue life or strength reduced remarkably with a decrease in the test frequency.

## 1. Introduction

Osteosynthesis is one of the most common techniques in traumatology and orthopaedic surgery. As reviewed by Merolli [1], methods applied to the treatment of bone fractures have evolved through many changes over the last century, which is reflected in the substantial progress in osteosynthesis techniques and the creation of new metallic fixtures. A perfect metal fixture for osteosynthesis should be bioinductive or, at least, biocompatible and bioresorbable while ensuring the stable functional behaviour of osteosynthesis [2,3]. Since the main purpose of implants is to provide the necessary mechanical support only during the bone regeneration process, biodegradable metals shift the existing paradigm in orthopaedy and traumatology in the direction of temporary structures, including bone implants and cardiovascular stents [4,5,6,7]. Metallic biodegradable devices have been conceived to overcome the main limitation of permanent implants by combining the advantages of previous generations of implants and making use of the excellent mechanical properties of metallic systems and the ability of harmless biodegradation of biopolymers.

Magnesium alloys represent a modern class of biodegradable metallic materials that have gained increasing attention as a potential matrix material for biomedical applications [7,8,9]. Not only do Mg alloys exhibit excellent biocompatibility and natural biodegradability, they also exhibit low density (1.7–2.0 g/cm^3^) and strength in excess of 250 MPa. Notably, the elastic moduli of Mg-based alloys (40–45 GPa) are better compared to the stiffness of cortical bones (3–20 GPa) than those of conventional metallic materials used as permanent implants (c.f., ~200 GPa for stainless steel, ~230 GPa for cobalt-based alloys, and ~115 GPa for titanium alloys [10]), which helps to eliminate the stress-shielding effect hindering the healing process.

Among hundreds of Mg-based alloy compositions proposed for orthopaedic applications, tertiary Mg-Zn-X alloys are by far the most studied due to their outstanding properties profile, both in vitro and in vivo. Ca, Y, and Zr are by far the most popular additions to Mg–Zn systems when seeking a balanced combination of mechanical and biofunctional properties [11,12,13,14,15,16,17,18,19,20,21,22,23]. The commercial alloy ZK60 has been proven versatile enough for processing by various techniques, from conventional extrusion and rolling to a wide range of severe plastic deformation techniques, resulting in substantial microstructure refinement and the enhancement of tensile [24], fatigue [25] and corrosion properties [26,27,28]. One of the factors impeding the broader uptake of Mg alloys is their poor corrosion resistance, resulting in a faster-than-desired degradation rate in chlorine-containing solutions, including human body fluids [29,30,31]. Discussing the corrosion behaviour of the alloy ZK60, Orlov et al. [24] noted that both Zn and Zr are more noble elements compared to pure Mg. Thus, they support cathodic reactions in the Mg–Zn–Zr systems at much greater rates and similar potentials. The presence of both Zn and Zr, either in solid solution or as intermetallic precipitates (such as MgZn_2_ or Mg_4_Zn_7_ and Zr_2_Zn_3_, commonly seen in ZK60) has been shown to promote cathodic kinetics and accelerate the corrosion rate [32]. While the corrosion properties, as well as the stress-corrosion cracking of the many Mg alloys, have been reasonably well understood, their corrosion fatigue performance has received only limited attention up to date despite its vital importance for many structural applications, including in biomedical fields [3,33,34,35,36,37,38,39,40,41]. Assessing the corrosion fatigue performance is complicated by many intrinsic and extrinsic factors that affect the fatigue life to a greater or smaller extent, which are to be taken into account. The extrinsic factors, besides the test frequency and specimen dimensions, include (but are not limited to) the environment, pH value and its variation throughout the test, temperature, and time of pre-exposure to corrosive media before the fatigue tests start. The ASTM standard [42], regulating the corrosion fatigue testing procedures for metallic implant materials, has been developed for permanent structures. It recommends that the testing frequency be set at 1 Hz. Considering that the ideal implant for osteosynthesis is supposed to maintain structural integrity in the human body for about 12 weeks, the 1 Hz frequency is too low for testing up beyond 10^7^ cycles. Therefore, fatigue tests are most commonly carried out at frequencies ranging from 10 to 100 Hz, and the effect of frequency has yet to be quantified. Moreover, it is important to notice that the thickness of many implants, like those used in maxillofacial surgery, is generally limited to approximately 2 mm or even less. Although the fatigue test standards require specimens with larger dimensions, in the present work, we confine ourselves to the sub-size specimens to mimic the geometry of interest for medical devices.

The frequency effect on the fatigue life of Mg alloys in air is usually insignificant or even negligible unless the crack propagation stage is of concern. Excellent support for that is provided by Nový et al. [43], who investigated the high and very-high cycle fatigue properties of extruded AZ31, AZ80, and ZK60 magnesium alloys tested at the two significantly different frequencies of 30 and 20 kHz. The smooth transition of the S-N curve was observed when the test frequency was drastically changed. A similar conclusion has been drawn recently by Liu et al. [44] on the example of hot extruded ZK60. The low-frequency sensitivity of fatigue strength in air reflects the generally relatively low strain rate sensitivity of the flow stress in Mg alloys at room temperature [45,46]. Let us notice that a common slight trend does exist towards increasing strength (both the yield stress and ultimate tensile strength) and decreasing ductility with increasing strain rate [47]. However, the strain rate sensitivity grows sharply with increasing temperature, as has been shown in [48] for several Mg-based alloys.

Since corrosion is a damaging process evolving in time, the corrosion fatigue life depends strongly on the time of exposure to the corrosive environment. It is, therefore, dependent on frequency and (to a lesser extent) on the wave shape of the loading cycles. Moreover, it depends also on the specimen’s cross-section since smaller specimens are more sensitive to corrosion-induced surface damage and local stress risers associated with corrosion pits and grooves. Considering that the development of corrosion-induced damage depends strongly on the chemical and electrochemical interactions of individual alloy elements with a specific environment, it is hard to compare the results of cyclic testing obtained at different frequencies, corrosive environments, and sample dimensions.

Since fatigue is essentially a surface-related phenomenon, the quality of the surface finish has a significant effect on the fatigue life of structural materials, which is larger at a larger number of cycles to failure [49,50,51]. Nevertheless, the significance of surface quality is, however, often overlooked and the details of the surface preparation are often missed in the reports due to the rapid propagation of corrosion damage, which, first and foremost, affects surface homogeneity.

Coatings are widespread protective solutions across many industries facing challenges with corrosion-related issues. A variety of coatings developed for biomedical devices have been reviewed in [52,53,54] and recently in [55]. While many functional properties of specific coatings have been addressed in these comprehensive reviews, corrosion fatigue remains the least covered. Zeng et al. [56] have pointed out that the influence of coatings on the fatigue life is not clear, although the overall positive influence of coatings on the corrosion fatigue life has been documented by many authors (see, e.g., [57]).

Thus, to partially reduce the existing shortage in corrosion fatigue data for Mg alloys, in the present work, within the stress–life-based approach, we analyse the cyclic fracture behaviour of the commercially available Mg-Zn-Zr alloy ZK60 in NaCl-containing simulated body fluid (SBF). Focusing on the effect of frequency on fatigue life, the influence of surface roughness and a thin Zr coating are assessed as well. For the sake of conservativism, (i) the sub-size specimens with the dimensions resembling those required for orthopaedic devices are tested, and (ii) the alloy ZK60, with very modest corrosion resistance to body fluids, has been chosen as a test material.

## 2. Materials and Methods

In this study, the hot-extruded commercial alloy ZK60 has been chosen as a representative of a class of relatively high strength biodegradable alloys [54] with low cytotoxicity [17,21,58]. Even though the alloy ZK60 does not pretend to be corrosion-resistant [59], it showed a better performance in Hank’s SBF compared to the popular WE43 or AZ91 alloys [58]. The chemical composition of the alloy was evaluated by the optical emission spectrometer ARL 4460 OES (Thermo Fisher Scientific, Waltham, MA, USA) and is given in Table 1.

The tensile tests were carried out using a screw-driven H50kT (Tinius Olsen, Redhill, UK) testing machine with a 50kN load cell and a clip-on extensometer at the nominal strain rate of 1 × 10^−3^ s^−1^ (as per the recommendation of the ASTM E8/E8M—21 standard [60]). Mechanical properties in air were obtained by testing the standard specimens with a round cross-section of 5 mm diameter and 20 mm gauge length.

For the purpose of the present work, miniature hour-glass specimens with a 2 × 2 mm^2^ cross-section were shaped by spark erosion from the as-extruded rods in the longitudinal direction (see [61] for details of the specimen geometry, dimensions and axial alignment in the grips). One part of the specimens was gently mechanically ground with sandpaper down to #2500-grade, while the other was further polished with diamond pastes down to a 0.25 μm particle size to a mirror-like finish.

The Electropuls E1000 (Instron, Norwood, MA, USA) electro-mechanical testing machine was used for high cycle fatigue (HCF) testing under load control mode in dry air at ambient temperature and in Ringer’s solution containing 8.6 g/L NaCl, 0.3 g/L KCl and 0.25 g/L CaCl_2_. The fatigue tests were performed according to the ASTM E466 standard [62] in the symmetric sinusoidal push-pull cycling (R = −1) mode with 10, 20, 40 and 80 Hz frequencies. Non-conductive grips were used to avoid galvanic coupling with the frame. A home-designed acrylic chamber was assembled on the testing machine so that the gripped sample was immersed in the circulating Ringer solution, stablised at 37 ± 0.5 °C. The testing cell was connected to the bath containing 10 L of the solution, which was continuously pumped through the cell at a rate of 0.2 L/min. The fatigue tests started immediately after submerging the specimen into the testing solution.

During the test, the pH value was continuously measured by the I160-MI digital pH-meter (Measuring Technique Ltd., Moscow, Russia) and corrected during the test to be at 7.4 ± 0.4, as recommended by the ASTM NACE/ASTM G31-12a standard [63]. The pH correction solution contained 500 mL of Ringer’s solution and 0.5–1 mL of phosphoric acid.

After the fracture in corrosive media, the specimens were immediately immersed in the standard 20%CrO_3_ + 1%AgNO_3_ aqueous solution for 1 min to remove corrosion products from the surface. For the fractographic examinations and roughness measurement, the confocal laser scanning microscope LEXT OLS4000 (Olympus, Tokyo, Japan) was used with 20× and 50× lenses. The initial microstructure was investigated by a field emission gun scanning electron microscope (SEM) SIGMA (Zeiss, Jena, Germany) equipped with a Hikari electron backscattering diffraction (EBSD) camera (EDAX/TSL, Mahwah, NJ, USA) and the orientation image microscopy software package OIM-6.2 from the same company. The EBSD maps were obtained from the regions of 160 × 160 μm^2^ with a step size of 250 nm. The indexed points, with a confidence index less than 0.1, were ignored in post-processing. The grains were identified using a minimum misorientation angle of 5°, and the grain diameter was determined from the grain area, assuming a spherical grain. For the grain identification procedure, the criterion for reliability was used with not less than 6–8 points with co-directional orientation within 5 degrees of misorientation [64].

In an attempt to deepen understanding of the effect of the surface on the corrosion fatigue of Mg alloys, thin (of 0.8 ± 0.2 μm) Zr-coatings coatings were applied to a bunch of polished specimens by ion-plasma sputtering in vacuum using a modified Bulat-6K (NSC KIPT, Kharkiv, Ukraine) system. The samples were preliminarily degreased in an ultrasonic bath with the Nefras solution. Immediately before being placed in the vacuum chamber, they were quickly cleaned with distilled water using a steam jet at 6 atm and 120 °C. To improve the adhesion of the coating, the glow discharge treatment was applied in an argon atmosphere at 1 Pa for 10 min prior to sputtering. The deposition process was carried out from one side-mounted cathode in argon at 0.0066 Pa. High-purity (99.99%) zirconium was used as a target. To ensure the uniformity of the coating over the entire specimen surface, the specimens were placed in the holder rotating at 20 rpm.

## 3. Results

### 3.1. Initial Microstructure and Tensile Properties in Air

The microstructure and crystallographic texture of the commercial hot-extruded ZK60 alloy has been well reported in abundant literature and well understood in the dependence on the parameters of extrusion, casting condition, and alloy treatment [18,65,66,67,68].

**Figure 1:**
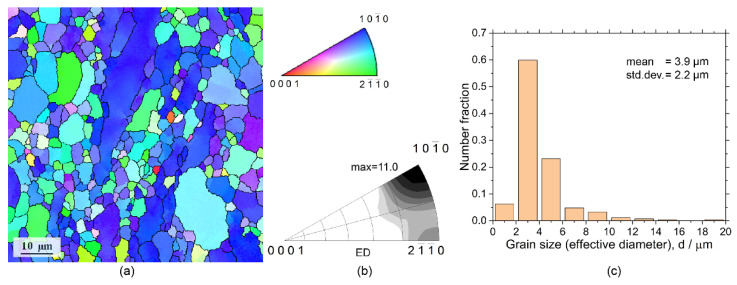
SEM-EBSD grain map (**a**) representing the typical microstructure of ZK60 alloy in the extrusion direction (ED), a crystallographic triangle with the inverse pole figure (IPF) colour codes applied to the grain map in (**a**) and the IPF (**b**), and the corresponding grain size distribution (**c**).

As it is commonly seen in the transverse sections of the ex-extruded ZK60 alloys (Figure 1), the microstructure is not uniform due to incomplete dynamic recrystallisation occurring during hot extrusion. Some coarse unrecrystallised grains are surrounded by fine equiaxed recrystallised grains, representing a typical extrusion microstructure. From the EBSD grain map and the corresponding distribution of grain sizes shown in Figure 1a,c, respectively, the mean grain size (edge grains exclusive) is about 3.9 μm, and the standard deviation is 2.2 μm.

Wrought Mg alloys usually generate a strong fibre texture during hot extrusion, depending on the alloy composition and extrusion parameters [69,70,71,72]. Figure 1b shows the inverse pole figures (IPFs) in the extrusion direction, exhibiting the commonly observed $\langle 10\bar{1}0\rangle$ fibre texture with the maximum texture index of 11 and the basal plane and $\langle 10\bar{1}0\rangle$ direction being preferably parallel to the extrusion direction (ED).

The second phases (usually MgZn and MgZn_2_) and their morphology and distribution in the cast and extruded ZK60 alloys have been well documented in abundant literature and, therefore, are not discussed here.

The tensile conventional yield stress determined at 0.2% plastic strain, ultimate tensile strength and the elongation to failure, determined from a set of three independent measurements, are 318 ± 3 MPa, 341 ± 2 MPa and 14 ± 2%, respectively.

### 3.2. Effect of Testing Frequency on Corrosion Fatigue

**Figure 2:**
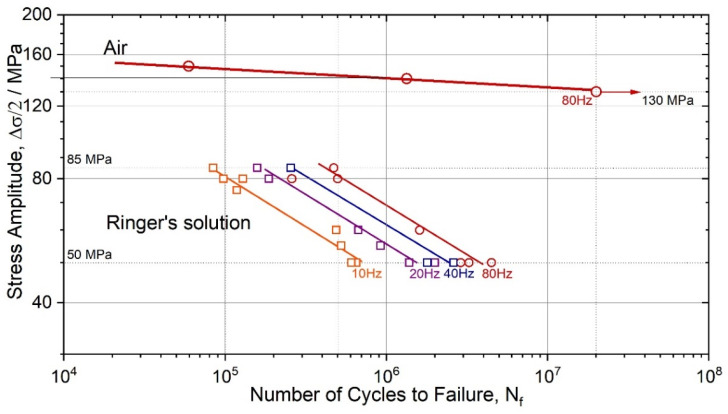
Wöhler plot for the ZK60 specimens with the ground surface tested at different frequencies—10, 20, 40 and 80 Hz in air and Ringer’s solution.

The bending fatigue strength (at 10^7^ cycles to failure and R = −1) of the wrought alloy ZK60 is typically ranged between 110–140 MPa [67], depending on the microstructure attained during processing (note that the axial fatigue limit can reach up to 150 MPa after grain refinement by severe plastic deformation [25]). The present results in air, Figure 2, fall nicely in the typical range: the endurance limit in uniaxial symmetrical push-pull testing is at 130 MPa (ground surface) in the high-cycle regime at *N_f_* = 2 × 10^7^ cycles to failure. After immersion in SBF, the fatigue strength dropped sharply from 140 MPa at 5 × 10^5^ loading cycles in air to 80 MPa in the solution at the same 80 Hz frequency. Such a significant degradation in mechanical performance is not unusual for Mg alloys. For instance, for the alloy AZ80 in 3.5% NaCl solution, the fatigue strength reduced by a factor of 1.4 and 2.6 at 10^6^ and 10^7^ cycles, respectively [73] (this is comparable to the present results although Al-containing AZ80 is nominally more corrosion-resistant than ZK60; moreover, the cross-section of the samples tested in [73] was five times larger than that in the present study, which provided higher mechanical stability of the specimen during the corrosion-induced surface degradation). With the reduction of frequency from 80 to 40, 20 and 10 Hz, i.e., with the proportionally increasing holding time in the corrosive solution, the deterioration of fatigue strength is even more pronounced, reducing further to 70, 65 and 55 MPa, respectively. Recently Han et al. [74] reported interesting results showing that low-frequency cyclic tension–compression alternating loads significantly accelerate the corrosion process in magnesium alloys, and the higher the applied frequency, the faster the corrosion. The trends observed in the present work are, however, different in that the lower the frequency and the longer the material is exposed to the aggressive solution, the greater the corrosion damage and the shorter the fatigue life.

### 3.3. Effect of Surface Roughness

**Figure 3:**
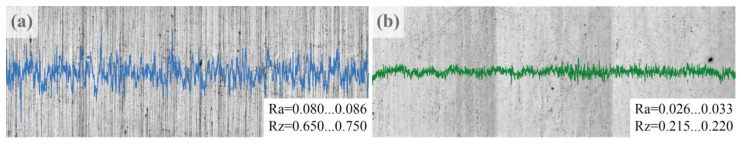
The surface appearance and the corresponding profiles after grinding (**a**) and polishing (**b**); roughness parameters are shown in the insets.

The surface roughness significantly affects the fatigue life. The fatigue testing standards ASTM E466 [62] and E606 [75] require final specimen polishing to impart a maximum surface roughness Ra of 0.2 μm. The typical profile of the samples prepared with two grinding options is shown in Figure 3. Measured surface roughness Ra and Rz values are shown in the insets. For both samples with different surface treatments, the Ra values are well below the acceptance threshold, albeit they differ by a factor of 3 or 4.

**Figure 4:**
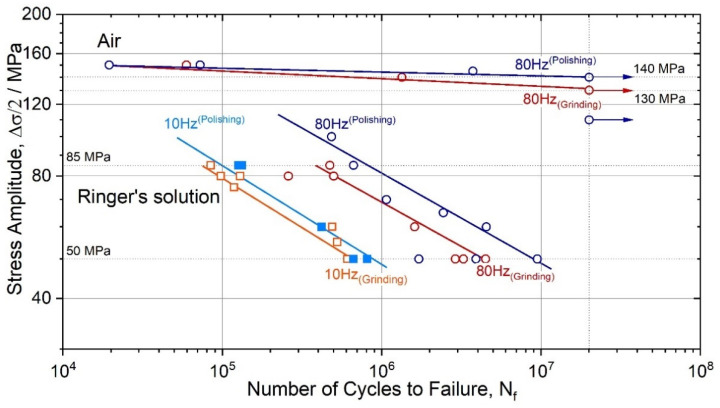
Wöhler diagrams for the ZK60 specimens with the ground and polished surfaces tested at 10 and 80 Hz in air and Ringer’s solution.

Compared to the ground specimens, the polished specimens tested in air at 80 Hz showed longer fatigue life. The endurance limit, corresponding to 2 × 10^7^ cycles to failure *N_f_* of polished specimens, was 140 ± 3 MPa, while their ground counterparts failed at the stresses by 10 MPa lower (Figure 4). The same trend is observed for the specimens tested in Ringer’s solution: polished samples had a consistently longer fatigue life at the same stress amplitude than their ground counterparts. Furthermore, one can notice that the difference in the tolerable stress amplitude for the polished and ground specimens increases with the test frequency in the corrosive solution.

### 3.4. Influence of Zirconium Coatings

As has been shown recently in [76], the deposition of the thin film of Zr on the surface of the specimen of the same ZK60 alloy reduced the corrosion rate in Ringer’s solution from about 13.8 to 10.5 mm/y when the coating thickness was 0.4 ± 0.2 µm and to 8.5 mm/y when the thickness of the protective coating was increased to 0.8 ± 0.2 µm. A significant (by orders of magnitude) reduction in the corrosion rate of AZ31 magnesium alloy has been observed after deposition of protective ceramic layers either by plasma electrolytic oxidation (PEO) [77,78] or atomic layer deposition techniques (ALD) [79,80].

**Figure 5:**
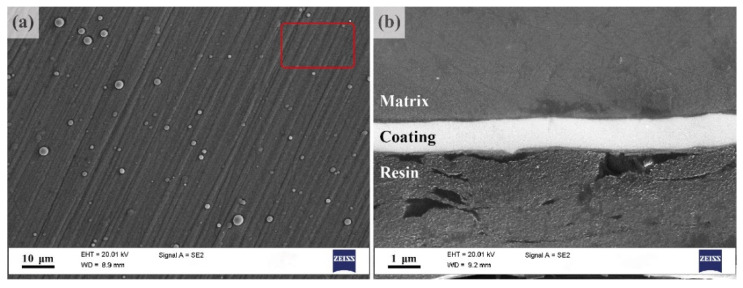
SEM images illustrating the Zr coating appearance of the specimen surface (**a**), and a view of the cross-section of the coating deposited on the ZK60 substrate (**b**); the area for the EDS spectroscopy analysis is indicated by a red rectangle (shown for the coating only).

The typical appearance of the surface of the coating applied to the fatigued sample is shown in Figure 5a, and the view of its cross-section is represented in Figure 5b. The coating is thin, of 0.8 ± 0.2 µm, dense, i.e., without visible voids, and it follows the surface waviness. Individual droplets of Zr of 1 μm diameter can be seen on the surface. The diffusion layer can hardly be formed under sputtering conditions, where the surface temperature did not exceed 200 °C, to preclude any microstructural modification of the substrate.

**Figure 6:**
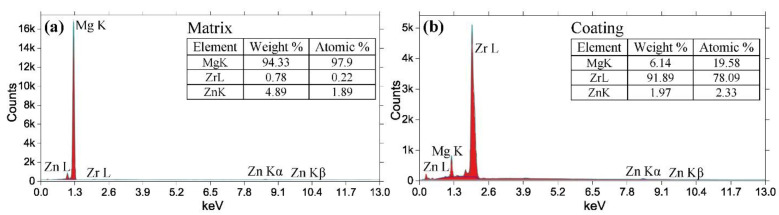
Energy dispersive X-ray (EDX) spectra obtained from the α-Mg matrix (**a**) and the zirconium coating (**b**) (the area for the analysis is indicated in Figure 5).

Results of the energy dispersive X-ray analysis carried out on the cross-section of the specimen (Figure 6) confirm that in line with the purpose of the present work, a reasonably pure Zr coating has been formed on the surface without oxidation.

**Figure 7:**
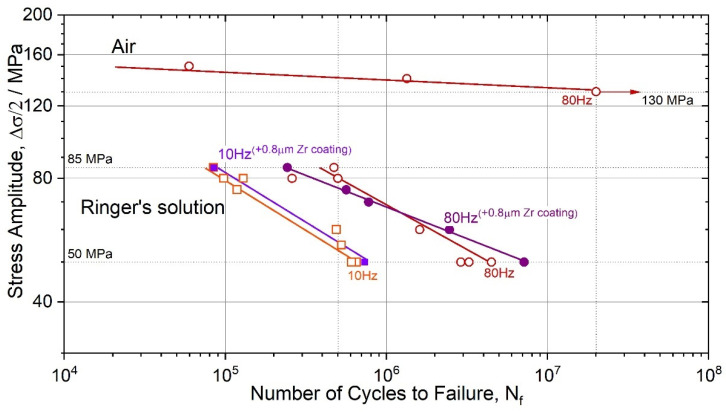
S-N plot for the ZK60 specimens—bare and coated by a 0.8 μm thick pure Zr coating—tested at 10 and 80 Hz in air and Ringer’s solution.

The endurance limit for the Zr-coated specimens was found to be 47 ± 3 MPa at *N_f_* = 10^6^ and 27 ± 3 at 10^7^ 10 Hz, and 68 ± 3 MPa and 48 ± 3 MPa at 80 Hz (Figure 7). Although the Zr coating of the same thickness helped to reduce the corrosion rate by at least a factor of 1.6 as assessed by hydrogen evolution after 110 h submersion in Ringer’s solution, the increase in the cyclic resistance was practically negligible.

## 4. Discussion

The corrosive environment ubiquitously causes a drastic reduction of the fatigue limit or the fatigue life at a given stress/strain amplitude [51]. On a general note, the most prominent contribution from corrosion to the initiation of fatigue cracks is attributed to surface damage created by corrosion. Corrosion pits are associated with local material loss due to anodic dissolution, which creates a strong stress riser. This is reflected by the drastic deterioration of fatigue life or strength, as illustrated by the S-N diagrams in Figure 2, Figure 4 and Figure 7. Corrosion pits are commonly blamed for the substantial degradation of fatigue performance of Mg alloys in a liquid environment [34,81,82]. The overall complexity of corrosion fatigue, in general, is amplified in magnesium alloys by the significant chemical reactivity of the alloy itself and the great variety of the deformation mechanisms involved. These mechanisms, in turn, depend on multiple metallurgical factors, such as texture and grain size [83,84,85,86].

**Figure 8:**
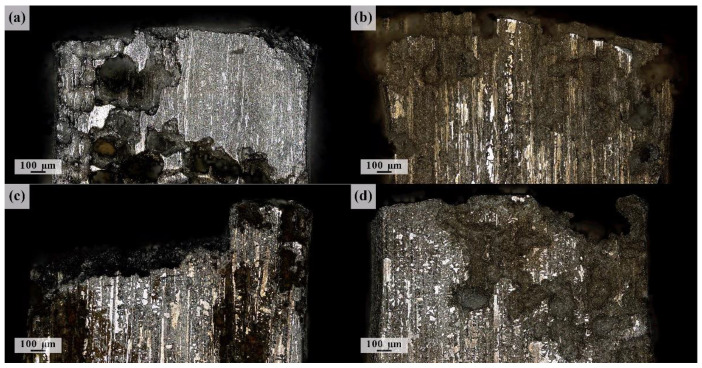
CLSM images of the side fracture surface of ZK60 specimens tested at 50 MPa stress amplitude after surface preparation by grinding (**a**,**c**) and polishing (**b**,**d**); (**a**,**b**) refer to the 10 Hz test frequency, (**c**,**d**) correspond to the 80 Hz frequency.

The typical appearance of the lateral surface of the specimens after fatigue fracture in SBF, followed by immediate removal of corrosion products, is shown in Figure 8. All specimens immersed in the corrosive solution suffered from multiple pits chaotically distributed over the surface. It is unclear whether the locations of individual pits are completely random or are pre-determined by the microstructural/chemical inhomogeneity. However, the results of Li et al. [87] show, quite unambiguously, that in line with the discussion given in the introduction, the micro-galvanic corrosion in the alloy ZK60 initiates primarily in the areas around the second-phase particles at the grain boundaries. Figure 8 indicates that once initiated, the corrosive pits tend to grow and deepen under the action of cyclic loads. This process, in turn, significantly accelerates the localised corrosion damage propagation by breaking the unstable layer of continuously forming corrosion products. No significant difference was found in the appearance of corrosion damage (in terms of geometry, depth and distribution of corrosion pits) on the ground and polished surfaces.

**Figure 9:**
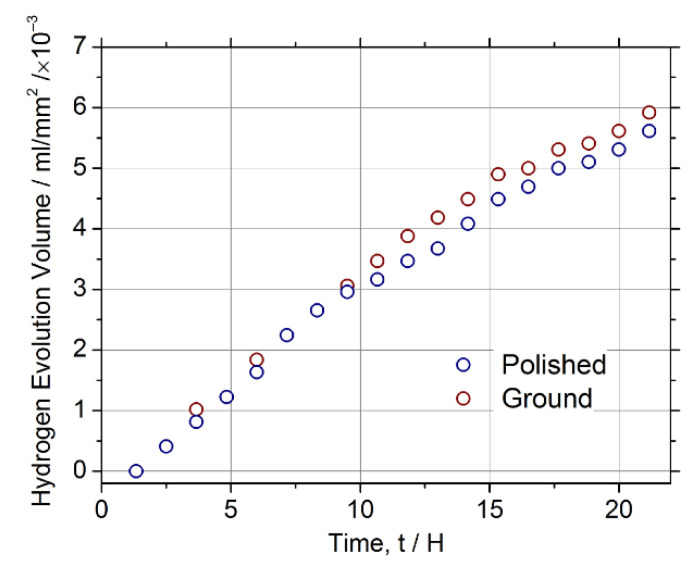
Hydrogen evolution curves for ZK60 specimens, with the ground and polished surfaces tested in Ringers’s SBF at 37 °C.

Similar conclusions are drawn from the measurements of hydrogen evolution in the same SBF carried out, as described in [88] according to the guidelines outlined in [89]. Figure 9 reveals no appreciable difference in the corrosion rate estimated during 20 h of testing of the polished and ground specimens.

Nonetheless, as it is commonly observed for fatigue tests in air [51], the increased surface smoothness improves the fatigue strength and fatigue life of Mg alloys. Contradictory results have been reported by Zeng et al. [56], who observed no improvement of fatigue resistance in NaCl solutions. In the present work, the polished specimens systematically showed a longer fatigue life when compared to their ground counterparts at the same stress amplitude, which was in line with the common trends seen in air.

**Figure 10:**
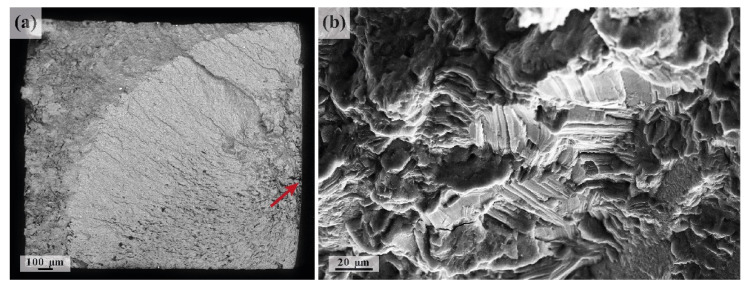
SEM fractography images of the alloy ZK60 specimen fatigued to fracture in air: (**a**) overview of the fracture surface (**a**) and a magnified view of the crack initiation site (**b**), pointed to by a red arrow in (**a**).

As has been discussed above, the collective action of fatigue and corrosion mechanisms leads to a significant reduction in the fatigue endurance of magnesium alloys, depending on the chemical composition and microstructure [90,91]. Figure 10 shows typical fracture surface morphology after corrosion fatigue of the specimens at 50 MPa stress amplitude. Since 50 MPa was the lowest amplitude applied, Figure 10 represents the most severe scenario of the corrosion impact on the fatigue damage propagation to failure. The most important observation is that the combined effect of the aggressive environment and cyclic stresses causes the accelerated loss of material from the surface exposed to the corrosive solution, resulting in significant surface roughening due to multiple corrosive pits and groves. As an immediate consequence of this effect, strong local stress risers form on the surface specimen. Due to cyclic loads amplified at these stress risers, the fatigue cracks propagate quickly inside the specimen, resulting in the increasing influence of mechanical factors such as notch sensitivity and stress intensity in fatigue development. As has been shown in [92], the significance of the microstructural factors concurrently reduces.

Characteristic regions corresponding to the different stages of a fatigue fracture in air are usually well-defined on the fracture surface, reflecting the crack initiation stage, followed by the area of steady crack propagation and the final rupture [25] (c.f., Figure 10). The river pattern, which is typical of fatigue, visible in Figure 10a, points to the region of crack initiation. The initiation stage I of the fatigue crack growth is known to be strongly microstructurally dependent [93,94]. It is controlled by the interplay between the dislocation slip along the basal planes and twinning in Mg alloys [26,82,95,96,97,98]. The traces of the slip planes, such as those visible in Figure 10b, are often seen in the fatigue fracture surfaces of Mg alloys at this stage (see, e.g., [99]).

**Figure 11:**
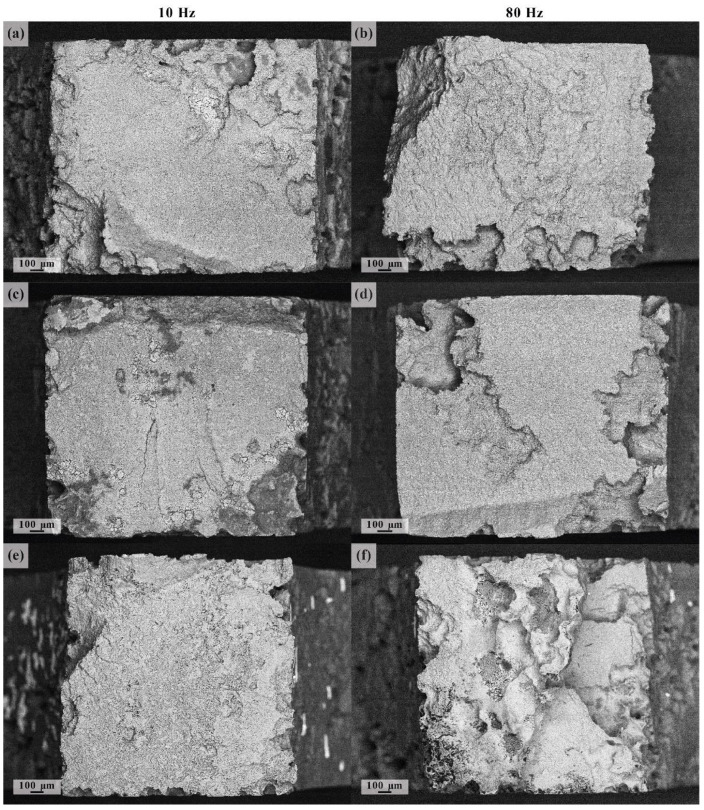
SEM images of the fracture surface after testing in Ringer’s solution with 50 MPa at 10 (**a**,**c**,**e**) and 80 Hz (**b**,**d**,**f**); initially ground surface (**a**,**b**), polished surface (**c**,**d**), and Zr-coated surface (**e**,**f**).

**Figure 12:**
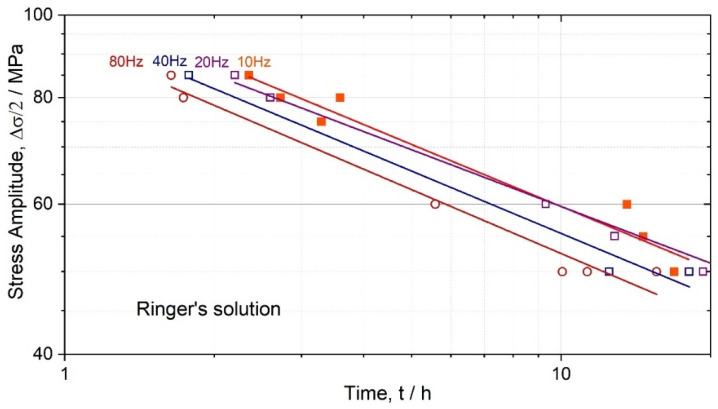
Fatigue diagrams for the ZK60 specimens plotted as the stress amplitudes vs. fatigue time, corresponding to different testing frequencies in Ringer’s solution.

On the contrary, the fractographic images of the investigated ZK60 specimens tested in SBF reveal (after removal of corrosion products) two notably different zones: one highlights severe corrosion-induced pitting along the perimeter of the fracture surface and the other the central flat matte areas with fine fibrous fracture morphology. Identifying the site (either single or multiple) of fatigue crack initiation is impossible for fracture reliefs of this kind. Neither it is possible to recognise the direction of the crack growth as there are no radial marks or visible roughness change due to crack propagation. Moreover, no regular striations are found on the fracture surface of the ZK60 specimens tested in the corrosive solution (Figure 11). Striations are characteristic of fatigue fracture morphology; they indicate successive positions of the advancing fatigue crack front in many structural materials [100], including Mg alloy fatigue tested under corrosion [91]). The presence of striations on the fracture surface is often regarded as a fingerprint of fatigue [101]. Striations form due to crack tip plasticity, the significance of which is characterised by the cyclic plastic zone forming at the crack tip as a result of unloading from the far-field load. If the size of the reversed plastic zone is too small, the striations may not form, and the fracture mechanism is a brittle one. On the one hand, the smaller the cyclic plastic zone, the lower the stress intensity ahead of the crack, and the slower the crack growth rate. On the other hand, the small plastic zone absorbs a smaller amount of mechanical energy needed for the crack advance, i.e., the crack propagation requires less energy. The small plasticity and the small, reversed plastic zone are reasonably anticipated when the stress amplitudes are small (e.g., 50 MPa or so, as in the present case) in corrosion fatigue testing. Therefore, it is not surprising that the conventional striations do not form, and the fine fibrous relief prevails. Even though the absence of striations does not exclude fatigue as a cause of failure [102], it strongly suggests that other factors play an important, if not decisive, role in the cyclic stress–corrosion damage evolution. Admittedly, the fractographic observations indicate that it is corrosion that takes the lead in mechano-chemical interactions and, finally, in the integrity degradation of small-size Mg alloy specimens under cyclic loads. However, if one assumes that the failure in SBF is governed chiefly by the adverse effect of corrosion, then the damage-controlling factor would be the holding time in the solution. In that case, the experimental data obtained for different test frequencies and plotted as a function of time to failure must converge to the same line. Corresponding plots are shown in Figure 12. The straight lines for different test frequencies converge to some extent; however, they do coincide only for 10 and 20 Hz frequencies. Consequently, corrosion damage, although having a crucial impact on durability, is not the sole factor controlling the failure process during fatigue testing of the Mg alloy in SBF.

**Figure 13:**
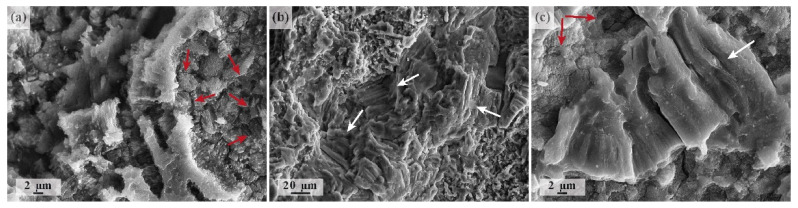
Enlarged fragments of the fracture surface after corrosion fatigue testing of ZK60 samples to failure at 50 MPa nominal stress amplitude and 10 Hz frequency, showing sporadically observed features that are typical of SCCs in many Mg alloys (including the same ZK60 [105]); red arrows point to the features characteristic of intergranular brittle fracture, and white arrows point to the fragments with a fluted fracture surface.

The reduction of the cross-section area caused by the corrosion attack from the specimen periphery is noticeable in Figure 11. With the ultimate tensile strength in excess of 300 MPa for the alloy ZK60, it is, however, not large enough to cause an ordinary quasi-static failure under tensile stresses as small as 50 MPa, corresponding to the lowest stress amplitude in the cyclic test. Neither does the fracture surface bear any similarity with the dimpled surface commonly observed after ductile fracture in tension [24]. Nevertheless, one can notice that the flat, fibrous fracture surface topology bears a lot of similarity to that reported for magnesium alloys fractured in physiological solutions under stress corrosion cracking (SCC) conditions (e.g., AZ91D [103,104] or AZ31 and ZK60, as shown by some of the present authors in dedicated investigations [28,105,106]). Figure 13 illustrates some of the observed features, which are typical of SCC in many Mg alloys (including the same ZK60 [28,105,106,107,108]) and are not seen in the specimens conventionally fatigued in air—there are visible signatures of intergranular brittle fracture (marked by red arrows) and multiple secondary cracks—and the characteristic fluted relief (marked by white arrows), which is indicative of SCC-induced plasticity ahead of the crack tip (flutes represent specific elongated dimples formed when voids are nucleated along slip-band intersections [109]). Of course, these features, which are sporadically observed on the fracture surface of the specimens fatigued in corrosive media, do not represent the predominant fracture mechanism, but rather, they indicate the plurality of the mechanisms involved in the fracture processes. The relatively simple picture of a fatigue fracture in air, represented in Figure 10, is exacerbated by both corrosion and SCC phenomena. The interaction of corrosion fatigue and stress corrosion cracking is a complex process that has not been understood to date and requires further investigation.

The transition from corrosion pits to fatigue cracks depends on the pit depth and shape and the corresponding local stress-strain conditions, as discussed in detail by Lynch [109,110,111]. The applied far-filed stresses are considerably amplified at the root of the corrosive pit, promoting premature fatigue crack initiation. It is timely to recall that SCCs can occur at stress risers even at remarkably low nominal stresses. The sharp fatigue cracks, emanating from the roots of the pits, increase the local stresses up to the values sufficient for SCC initiation. Thus, SCC processes can co-exist with the fatigue processes, at least at the relatively slow stage of initiation of the final fatigue crack, as will be unfolded further below.

**Figure 14:**
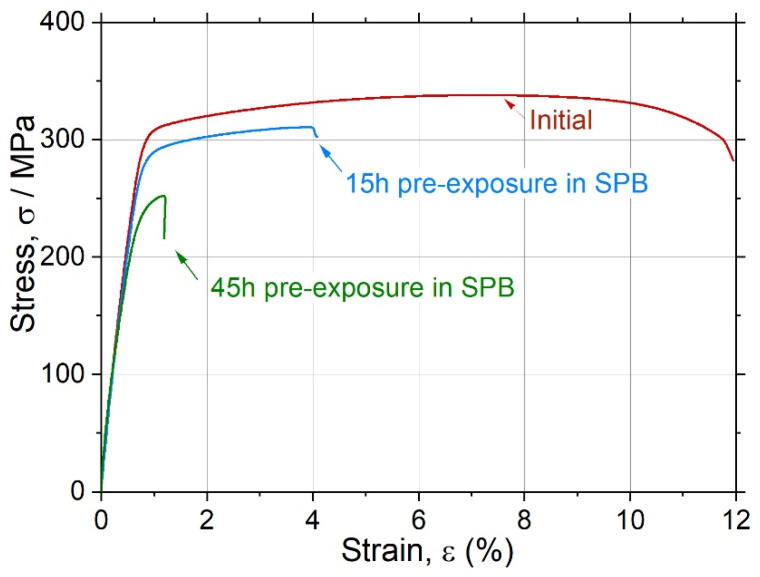
Engineering tensile stress–strain curves the ZK60 specimens tested in air in the initial condition and after pre-exposure in Ringer’s solution for 15 and 45 h.

To get a deeper understanding of the impact of conventional corrosion on the mechanical response, additional quasi-static tensile tests were performed on the miniaturised specimens (20 × 2 × 2 mm^3^) with the same cross-section area used for the fatigue testing campaign. The specimens were pre-immersed to Ringer’s solution for 15 and 45 h, which correspond to the average and longest time to fatigue failure, respectively, at the stress amplitude of 50 MPa in the same environment. After pre-exposure, the specimens were cleaned with isopropanol, dried by compressed air and immediately mounted in the grips of the tensile machine. The time lag between the end of pre-exposure in SBF and testing was about 15 min. These tests were performed in air at the strain rate of 4.2 × 10^−3^ s^−1^ with the 5 kN load cell. Results are shown in Figure 14. Although the ultimate tensile strength reduced from 338 MPa to 310 and 252 MPa after 15 and 45 h pre-immersion times, respectively, it still remained high—much higher than the fatigue fracture stress observed at the same testing time under cyclic loading. However, the ductility loss was notably more severe—from 12% to 4% and 1%, respectively, which is indicative of the embrittlement associated with SCC.

**Figure 15:**
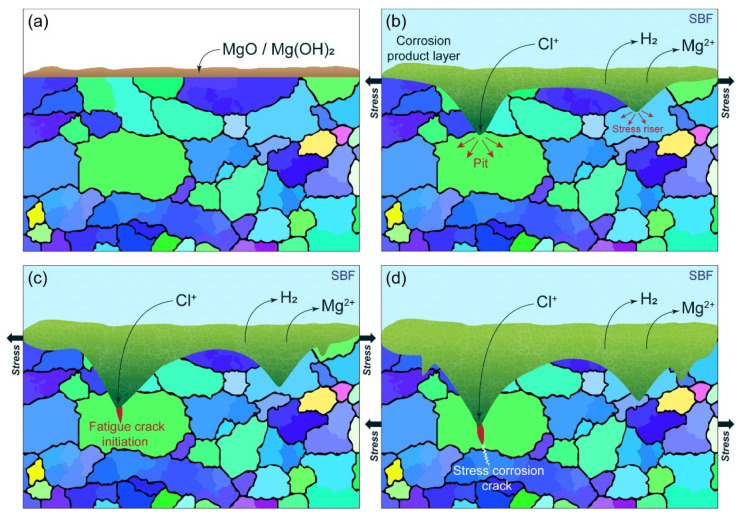
A simplified schematic illustration of the corrosion-fatigue-induced damage propagation. (**a**) Initial state with the MgO/Mg(OH)_2_ protective film formed on the surface; (**b**) advanced stage of the stress-stimulated corrosion process with the protective film broken, resulting in the formation of corrosion pits and the corrosion product layer; (**c**) initiation of the fatigue crack at the stress concentrator created by the notch-like root of the corrosion pit; (**d**) stress corrosion cracking initiated in the vicinity of the sharp fatigue crack.

The scenario of the stress-corrosion fatigue-induced multistep fracture process envisioned from the results of the present study is schematically outlined in Figure 15. The MgO/Mg(OH)_2_ protective film (a) forming on the surface of the Mg alloys in a neutral solution containing Cl^−^ ions breaks locally under the action of cyclic stresses, providing the aggressive environment access to the bare metal surface. The corrosion pits nucleate and grow both laterally and in depth throughout the duration of the test, providing significant stress concentration at the roots of multiple pits (b). The fatigue cracks initiate in the vicinity of the pits (c) at nominal stresses, which are remarkably smaller than those required for fatigue crack initiation in smooth bodies in the air of the inter environment. Corrosive products forming at the bare surface facilitate the occurrence of stress-corrosion cracking (d), which can become either a prevailing fracture mode or develop concurrently with fatigue processes. It would be naïve, of course, to believe that the proposed picture matches reality comprehensively and that the scenario illustrated by Figure 15 is the only viable one. As has been mentioned above, the details of the fatigue and SCC interactions are not clear from either a microstructural or mechanistic viewpoint. It, however, simply and consistently explains why it is practically impossible to find the unique site of fatigue crack initiation, why the fatigue striations are not observed, and why the SCC features can be recognised on the fracture surface.

As a final note, orthopaedic implants are supposed to maintain their mechanical integrity for at least 5 × 10^5^ loading cycles in body fluids (see, for example, [34,35,112]). This is, of course, a very approximate number, which, however, can be used as a reference point for practical guidance. The alloy ZK60 used in the present study possesses the fatigue strength ranging from 55 to 80 MPa in Ringer’s SBF at 5 × 10^5^ cycles, depending on the testing frequency. The demonstrated fatigue resistance is higher than the bone fatigue strength, which is in the order of 23–30 Mpa [34]. This result may suggest that ZK60, despite its relatively low corrosion resistance and high susceptibility to SCC, still meets the requirements imposed on the corrosion fatigue properties for orthopaedic implant applications. The use of the miniaturised specimens for fatigue testing makes this statement more conclusive.

## 5. Concluding Remarks and Outlook

The present work stands in the backdrop of studies similarly demonstrating the significant reduction of the fatigue performance of Mg-based alloys in corrosive media containing chlorine ions, e.g., body fluids. The results of the present work suggest that accelerated environmentally assisted degradation under cyclic loading occurs in magnesium alloys due to a complicated interplay between corrosion damage, stress corrosion cracking and cyclic loads. The occurrence of corrosion fatigue in Mg alloys is exaggerated by the strong sensitivity to the testing frequency. The fatigue life or strength reduced remarkably with the decrease in the test frequency, i.e., with the increase in the time of exposure to the aggressive environment. Thus, the present results highlight an exciting area of research that lies in the interaction between corrosion, stress-corrosion cracking and cyclic loads. It is fair to say that the mechanisms for these interactions are not well understood, which calls for more research on the topic. Of particular interest is low-frequency fatigue testing, which is practically important and might be helpful to shed light on the aforementioned interactions. Moreover, interrupted cyclic tests, followed by detailed microstructural surface examinations, have to be carried out to this end, and this is a scope of our future studies.

Even though a surface treatment by fine mechanical polishing or applying a thin metallic biocompatible coating can help to improve the poor protective capacity of the passive layer on the magnesium surface and reduce the average corrosion rate, it does not warrant the equivalent improvement in corrosion fatigue resistance. Hence, there is still a lot to desire to improve the practicality of surface treatment procedures that can be applied to Mg alloys aimed at functional biomedical applications. The somewhat negative message delivered in the present communication is, however, partially compensated by the observation that even for miniature specimens, which particularly suffer from corrosion attack, the desired corrosion fatigue resistance can be achieved, in principle, with the use of relatively high-strength alloys. Thus, tailoring microstructures of biocompatible magnesium alloys towards obtaining a higher strength, preferably with enhanced ductility and improved corrosion resistance, remains topical and challenging in spite of extensive research efforts in recent decades. Paired with the informed choice of chemical composition, the microstructure refinement via severe plastic deformation techniques can be a viable strategy that is worth exploring in conjunction with a wealth of existing surface treatment technologies.

As a final remark, we should notice that the in vitro biocorrosion behaviour of magnesium alloys, in general, and of ZK60, in particular, depend strongly (besides on frequency) on the chemical composition of SBFs, which vary greatly in the concentration of primary ions such as Mg^2+^, Na^+^, K^+^, Ca^2+^, Cl^−^ phosphates and carbonates/hydrocarbonates and may include cells, proteins and/or organic compounds found in blood plasma [113]. As reviewed by Esmaily et al. [31], the exact roles of the different solutes present in various SBFs in the Mg dissolution mechanisms have not yet been unambiguously clarified. However, it is clear that different corrosion products form on the surface, depending on the chemical composition of the solution: compared to simple saline solutions, more complex corrosion products, including different types of (Ca/Mg) phosphates and carbonates, form in SBFs [114]. Thus, understanding the mechanisms through which biofunctional properties of Mg alloys in specific solutions mimicking human body fluids can be affected by processing will pose new interesting scientific problems and challenges to be addressed in future research.

## Figures and Tables

**Table 1:** Chemical composition of the investigated alloy (in wt. %).

Mg	Zn	Zr	Al	Fe	Cu	Ni	Mn	Ce	Nd	Si
Bal.	5.693	0.860	0.001	0.004	0.002	0.004	0.005	0.027	0.062	0.008

## Data Availability

Data are available from the corresponding author upon reasonable request.
